# High glucose level and angiotensin II type 1 receptor stimulation synergistically amplify oxidative stress in renal mesangial cells

**DOI:** 10.1038/s41598-019-41536-z

**Published:** 2019-03-26

**Authors:** Tetsuya Akaishi, Michiaki Abe, Hiroshi Okuda, Kota Ishizawa, Takaaki Abe, Tadashi Ishii, Sadayoshi Ito

**Affiliations:** 1 Department of Education and Support for Regional Medicine, Tohoku University Hospital, Sendai, Japan; 2 Division of Nephrology, Endocrinology and Vascular Medicine, Tohoku University Hospital, Sendai, Japan

## Abstract

Oxidative stress in renal mesangial cell causes diabetic glomerular changes. High glucose levels and angiotensin II (Ang II) are known to stimulate superoxide production in renal mesangial cells. However, it has been unclear whether Ang II stimulation and pre-conditioning with high glucose affects the same pathway of superoxide production in renal mesangial cells or not. In this study, we examined the levels of oxidative stress under Ang II stimulation in renal mesangial cells preincubated for six hours at various glucose levels. Intracellular levels of reactive oxidative species (ROS) were measured using dihydroethidium or 5′,6′-chloromethyl- 2′,7′ dichlorodihydro-fluorescein diacetate, which facilitates the detection of intracellular ROS under real-time fluorescent microscope. Ang II-induced elevated intracellular ROS levels were detected only when the cells were pre-incubated with high levels of glucose (13.5 mM, 27.8 mM), but was not detected under normal glucose condition (5.5 mM). Production of Ang II-induced intracellular ROS was higher under pre-treatment with 27.8 mM glucose compared to pretreatment with 13.5 mM glucose level. This ROS production in mesangial cells was induced within several minutes of the initiation of Ang II stimulation under high glucose levels. The production of intracellular ROS was significantly reduced in the presence of angiotensin II type1-receptor (AT1R) antagonist, whereas it was augmented in the presence of angiotensin II type2-receptor antagonist. In conclusion, Ang II-induced oxidative stress was augmented by high glucose levels and ROS levels were further alleviated in the presence of AT1R antagonists.

## Introduction

Renal glomerular changes in diabetic patients are recognized as mesangial expansion and fusion of foot processes on podocytes^1,2^. These glomerular pathological changes are triggered by oxidative stress and one of the generator of such changes is mesangial cell (MC)^3,4^. MCs are known to produce extracellular matrix as supportive tissue and to maintain glomerulocapillary structure by contracting, or to act as immune cells like phagocytes^5–7^. Since MCs in renal glomeruli can contract and hold the capillaries together at the stalk, damages in them could result in mesangial expansion. Previous studies on the pathophysiological mechanism in diabetic nephropathy reported that very high concentration of glucose (*i*.*e*. ~25–30 mM), which is equivalent to about 450–540 mg/dL serum glucose level, increases the oxidative stress via increase in reactive oxygen species (ROS) in MCs^8,9^. However, whether such intracellular ROS production also occurs at lower levels of glucose (10–15 mM; 180–270 mg/dL serum glucose level) is still inconclusive. Since microalbuminuria, which is associated with diabetic microvascular complication, can be observed in the early stage of impaired glucose tolerance, there is a possibility that such ROS production also occurs in this condition under mildly elevated glucose levels^10^. If such a speculation could be validated, it would strongly suggest that hyper-activated ROS production in renal mesangial cells are associated with the pathogenesis of diabetic glomerular injury from the early stages.

In addition to the high glucose levels, angiotensin II (Ang II) stimulation has been also reported to promote ROS production in MCs^11–13^. Only a few studies that evaluated the ROS production in MCs under different glucose levels and Ang II stimulation conditions, especially under mild glucose levels have been conducted. Moreover, there has been no study on the evaluation of ROS production in MCs under various glucose levels and Ang II stimulations given at different times.

To verify that ROS production in MCs, stimulated by elevated glucose levels and Ang II, is one of the essential pathophysiological mechanisms in diabetic nephropathy, we exposed the cultured MCs separately to mild glucose levels and Ang II stimulations at different timings and observed the ROS production in them over time. In this study, we measured the production of ROS in MCs over time after pre-incubating for six hours under various glucose levels (5.5, 13.5, and 27.5 mM) with or without Ang II stimulation. Besides, to investigate the synergistic negative effect of glucose and Ang II, Ang II was administered at different timings in the presence of glucose.

## Materials and Methods

### Reagents

Dihydroethidium (DHE) and 5′,6′-chloromethyl-2′,7′ dichlorodihydro-fluorescein diacetate (CM-H_2_DCFDA) were purchased from Molecular Probes Inc. (Eugene, OR). Both reagents were dissolved with DMSO and diluted for daily use. Modified Hanks’ balanced salt solution (HBSS) and fetal calf serum (FCS) were purchased from Invitrogen (Carlsbad, CA). D-glucose (glucose), L-glucose, Ang II, HEPES, L-arginine, Tiron, PD123319 (angiotensin II type 2-receptor antagonist), and polyethylene glycol (PEG)-catalase were purchased from Sigma (St. Louis, MO), while RNH-6270 (angiotensin type1-receptor antagonist) was purchased from Sankyo Co. (Tokyo, Japan).

### Mesangial cell cultures

The human mesangial cells (MCs) (Cambrex, Walkersville, MD) were spread and cultured on 15 mm-round cover glasses in MsBN medium (Cambrex, Walkersville, MD) containing 5% v/v FCS and 0.1% v/v gentamicin sulfate/amphotericin-B under an atmosphere of 95% O_2_/5% CO_2_ at 37 °C. The cells were used from 6^th^ to 10^th^ passage.

### Real-time fluorescence imaging of cells

Fluorescence measurements were obtained using IX71 inverted microscope (Olympus, Tokyo, Japan) attached with a 60× (numerical aperture 0.9) water-immersion objective lens as previously reported^14^. The signal was detected by a cool CCD video camera Ixon (Andor Co., Tokyo, Japan) coupled to Lambda-10.2 (Sutter Instruments, CA), and excitation was provided by Sutter DG-4 and 175-W Xenon-Arc lamp (Sutter Instruments, Navato, CA). For the experiments, cover slips were placed in an imaging chamber (RC-42LP and RC-43C; Warner Instruments, Hamden, CT) mounted on the stage of the inverted microscope and maintained at 37 °C with TC-344B thermo-warmer for chamber and TA-29 thermo-warmer for perfusate (Warner Instruments, Hamden, CT). The changes in fluorescence intensity were quantified using MetaFluor imaging software (Universal Imaging Co., Ypsilanti, MI) for each experiment. To exclude the possibility of selection bias of the measured MCs, the fluorescence intensity of all living mesangial cells in each cover glass was measured.

### Measurement of oxidative stress in mesangial cells

#### Protocol 1 (evaluation of O^.2−^ production in MCs under various conditions)

**Figure 1:**
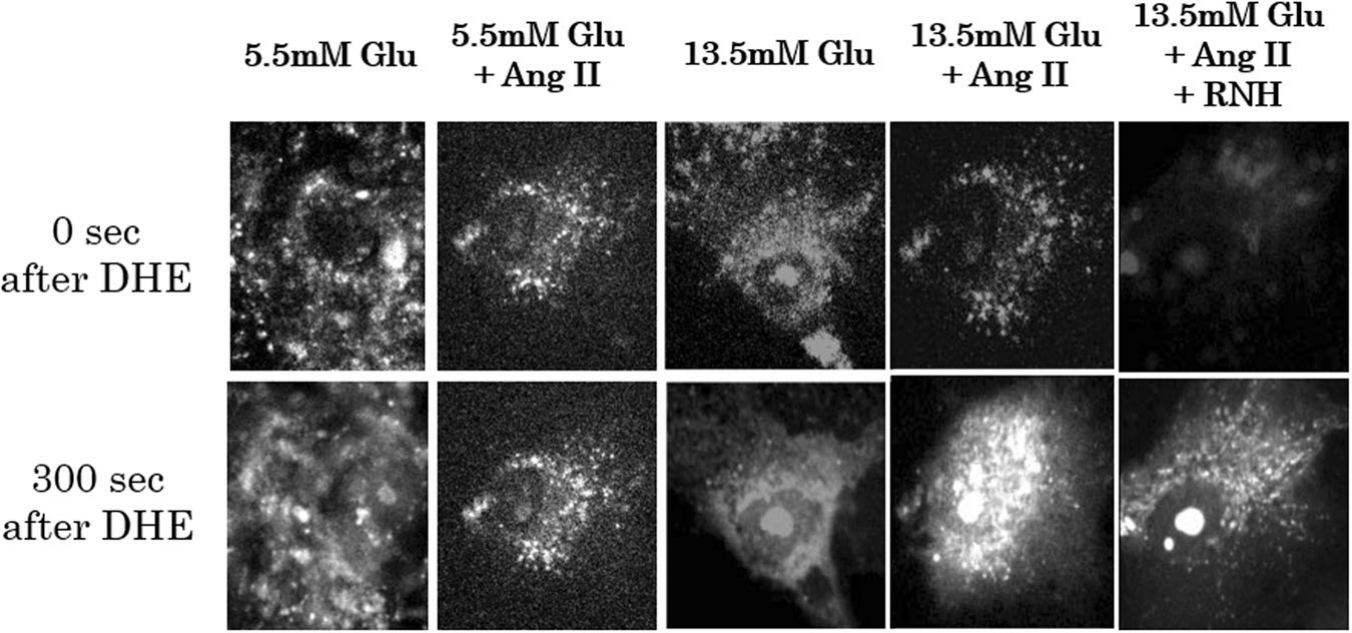
Fluorescence intensity of ethidium derived due to the oxidation of DHE by intracellular superoxide (Protocol 1). The fluorescence intensity reflects the levels of intracellular superoxide. The dihydroethidium (DHE) administered in the buffer penetrates cell membrane and is oxidized by superoxide anions to form ethidium (Eth), which emits red fluorescence (excitation filter: 480 nm wavelength; emission filter: 605 nm wavelength). All living cells, which were attached to the bottom of cover glass, were examined for the fluorescence emission after every 10 s. We estimated that the intracellular superoxide level was the highest after pre-conditioning with 13.5 mM glucose + Ang II. Abbreviations: Ang II, angiotensin II; DHE, dihydroethidium; Glu, glucose; mM, mmoL/L; RNH, angiotensin II type 1 receptor antagonist; s, seconds.

MCs cultured till ~75–90% confluence on 15 mm-round cover glasses were incubated in the culture media containing 5.5 mM (100 mg/dL) or 13.5 mM (240 mg/dL) glucose with or without 10^–7^ M Ang II for 6 hours. The cover glass was set on the chamber of the microscope and cells were washed with HBSS containing 20 mM HEPES, 5.5 mM glucose, and 100 μM L-arginine (pH7.4). The buffer was subsequently exchanged with assay buffer containing 5 µM of DHE. DHE-penetrated cell membrane and intracellular DHE is oxidized by O^.2−^ to form ethidium (Eth), which integrates into DNA. Intracellular Eth was excited at 480 nm wavelength and red fluorescent signal was detected through emission filter of 605 nm wavelength, as shown in Fig. 1^14–16^. As dying cells were detached from the bottom, imaging signal of Eth was collected after every 10 s from each living cell attached to the bottom of the cover glass. For each condition, a total of five cover glasses were prepared, each of which contained about 10–15 live MCs on the bottom. Fluorescent signal from each of all living MCs under each pre-condition and timing was quantified.

#### Protocol 2 (evaluation of acute response of H_2_O_2_ against Ang II stimulation in MCs)

MCs were incubated in culture media containing 5.5, 13.5, or 27.8 mM glucose for 6 hours. The cells were next washed with vehicle buffer containing 300 nM CM-H_2_DCFDA at a continuous flow rate of 1 mL/min. CM-H2DCFDA is converted to H_2_DCF by cellular esterase and trapped in the cell. Then, intracellular H_2_DCF is oxidized by ROS and gets converted into fluorescent DCF^17,18^. For stimulation of MCs, the assay buffer was exchanged with stimulation buffer containing 10^−7^ M of Ang II. The active form of DCF was excited at 480 nm wavelength and the signal was detected through emission filter of 535 nm wavelength. The imaging signals of cells attached on cover glass were collected after every 10 s.

### Statistical analysis

In protocol 1, the slopes of the Eth response were calculated from the final 300 s of experimental period after the control equilibration. In protocol 2, the slopes of the DCF were calculated from the final 150 s of vehicle and Ang II period. All data were presented as mean ± standard error (SE). The significance of slope in the responses between vehicle and compounds was performed using unpaired Student’s *t*-test. The significance of change in the slope of each response curve from the control period (0–160 s) to the experimental period (180–340 s) was evaluated using paired *t*-test.

## Results

### Synergistic effect of Ang II and glucose on superoxide anion production in MCs under chronic condition (Protocol 1)

**Figure 2:**
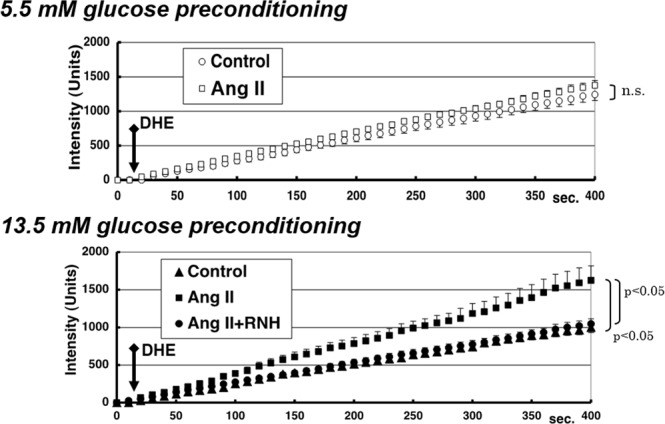
Chronological changes in levels of intracellular ethidium under different pre-conditionings (Protocol 1). The slope of intracellular ethidium signal from each living mesangial cell reflects the level of intracellular superoxide after each pre-conditioning (above: 5.5 mM glucose; below: 13.5 mM glucose). Ethidium signal was measured every 10 s after administering DHE. The signal slopes were significantly different in the presence of Ang II stimulation after pre-conditioning with 13.5 mM glucose, but were not different after pre-conditioning with 5.5 mM glucose. The error bar of each plot shows standard error of the measured ethidium signal. Abbreviations: Ang II, angiotensin II; DHE, dihydroethidium; n.s., not significant; RNH, angiotensin II type 1 receptor antagonist.

MCs were pre-incubated with 5.5 mM or 13.5 mM glucose, with or without the presence of 10^−7^ M of Ang II for six hours. Then, DHE was added and the intracellular Eth level was measured after every 10 s for up to 400 s. The chronological changes in intracellular level of Eth under such conditions are shown in Fig. 2. The production of Eth was significantly increased by pre-conditioning with 13.5 mM glucose along with Ang II stimulation, compared to that in control (slope: 4.24 ± 0.42 units/s; *p* < 0.05, compared to control); however, it was not increased after pre-conditioning with 5.5 mM glucose even with Ang II stimulation (slope: 3.57 ± 0.13 units/s; *p* > 0.05, not significant, compared to control). There was no increase in intracellular ROS level after pre-treatment with AT1R antagonist (RNH6720) and it was comparable with the ROS level in control (slope: 2.61 ± 0.16 units/s; *p* < 0.05, compared to Ang II stimulated samples).

Although not shown in Fig. 2, a scavenger agent of superoxide anions, Tiron (10 µM), ameliorated the Eth responses obtained after pre-treatment with 13.5 mM glucose along with Ang II stimulation, which confirmed that stimulation with high glucose along with Ang II increased the production of O^.2−^ in MCs (slope: 1.04 ± 0.45 units/s; *p* < 0.05, compared to samples treated with Ang II without Tiron condition). Furthermore, to exclude the effect of osmolarity due to high glucose, the substitution of buffer with 13.5 mM L-glucose did not enhance the Ang II-induced O^.2−^ production (slope: 0.93 ± 0.21 units/s; *p* < 0.05, compared to samples treated with Ang II along with 13.5 mM D-glucose).

### Effect of Ang II on ROS production in MCs (Protocol 2)

**Figure 3:**
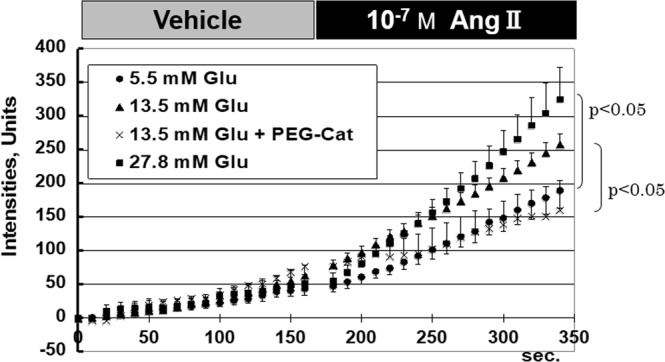
Chronological changes in levels of intracellular activated CM-H_2_DCFDA before and after angiotensin II stimulation (Protocol 2). In this protocol, acute response of H_2_O_2_ level against angiotensin II stimulation in mesangial cells was evaluated. The vehicle buffer containing only inactivated CM-H_2_DCFDA without angiotensin II did not increase the activated CM-H_2_DCFDA levels in any of the samples pre-conditioned with glucose. Once 10^−7^ M angiotensin II was added to the experimental buffer, intracellular superoxide level was rapidly enhanced within several minutes after changing the buffer at every glucose level, especially under high glucose levels (13.5 mM and 27.8 mM). The error bar of each plot shows standard error. Abbreviations: Ang II, angiotensin II; Glu, glucose; mM, mmoL/L; PEG-Cat; polyethylene glycol catalase; s, second.

Effect of Ang II on the production of ROS was assessed in MCs. MCs were pre-incubated in the medium containing 13.5 mM glucose for 6 hours. After the pre-incubation, ROS produced in the MCs were quantified using CM-H_2_DCFDA. O^.2−^, a primary compound of ROS, rapidly catalyzes into hydrogen peroxide (H_2_O_2_) or reacts with NO to form peroxynitrite (ONOO^−^). H_2_DCF reacts rapidly with both H_2_O_2_ and ONOO^−^, and thus, reflects the total amount of O^.2−^, indirectly. As shown in Fig. 3, the vehicle buffer containing inactivated CM-H_2_DCFDA without Ang II did not increase the levels of activated DCF. However, once 10^−7^ M Ang II was added to the experimental buffer, the intracellular ROS level was significantly and rapidly increased (slope for 0–150 and 200–350 s: 0.38 ± 0.10 vs. 1.11 ± 0 .06, *p* < 0.05). Pre-treatment with 50 mU/L of PEG-catalase, which reduces oxidative stress, significantly reduced the slope (0.50 ± 0.08 units/s), compared to that in samples treated with 13.5 mM glucose without PEG-catalase (p < 0.05). These results suggested that the production of the intracellular ROS started immediately (within less than 100 s) after the exposure of MCs to Ang II stimulation.

### Effect of glucose concentration on the production of Ang II-induced H_2_O_2_ (Protocol 2)

We also compared the levels of Ang II-induced ROS production under various glucose levels (*i*.*e*. 5.5 mM, 13.5 mM, and 27.8 mM). MCs were pre-incubated in the medium containing various concentrations of glucose for 6 h. After the pre-incubation, the media were replaced with vehicle buffer. Then the vehicle was exchanged with the experimental buffer containing 10^−7^ M Ang II. If ROS is produced in the MCs, fluorescent signals of activated DCF get enhanced. As shown in Fig. 3, the vehicle buffer containing inactivated CM-H_2_DCFDA, without Ang II, did not increase the levels of activated DCF in samples pre-conditioned with any of the glucose concentrations (slopes: 0.28 ± 0.06, 0.38 ± 0.10, and 0.31 ± 0.06 units/s for 5.5 mM, 13.5 mM and 27.8 mM glucose, respectively). Interestingly, once 10^−7^ M Ang II was added to the experimental buffer, the intracellular ROS was significantly increased under every glucose concentration (slopes: 0.90 ± 0.13, 1.11 ± 0.06, and 1.70 ± 0.28 units/s for 5.5 mM, 13.5 mM, and 27.8 mM glucose, respectively). These data suggested that the level of Ang II-induced mesangial oxidative stress after Ang II exposure was dependent on the glucose level during pre-conditioning.

### Effect of two types of angiotensin II receptors on oxidative stress

**Figure 4:**
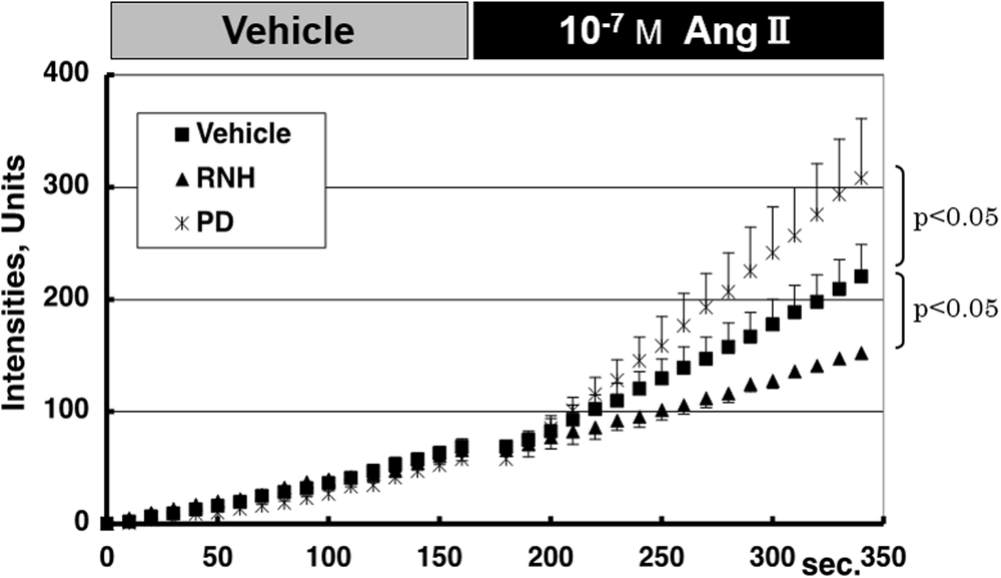
Chronological changes in levels of intracellular activated CM-H_2_DCFDA in the presence of angiotensin II receptor antagonists (Protocol 2). Acute response of H_2_O_2_ levels against angiotensin II stimulation in mesangial cells was significantly enhanced by administration of angiotensin II type 2 receptor antagonist (PD), but was significantly reduced by administration of angiotensin II type 1 receptor antagonist (RNH). Based on this result, angiotensin II-induced generation of oxidative stress in mesangial cells was suggested to be mediated by angiotensin II type 1 receptor. The error bar of each plot shows standard error. Abbreviations: Ang II, angiotensin II; M, mol/l; PD, angiotensin II type 2 receptor antagonist; RNH, angiotensin II type 1 receptor antagonist; s, second.

While the pre-treatment with RNH-6270 significantly suppressed the AT1R-induced oxidative stress in mesangial cells (slope: 0.53 ± 0.10 units/s; *p* < 0.05, compared to vehicle), pre-treatment with 10 µM PD123319 (PD), an AT2R-specific blocker, significantly enhanced oxidative stress (slope: 1.58 ± 0.22 units/s; *p* < 0.05, compared to vehicle), as shown in Fig. 4. These results show that ROS production is regulated by the balance of AT1R and AT2R.

## Discussion

In this study, we showed that Ang II-mediated induction of oxidative stress in mesangial cells is dependent to the extracellular glucose level around the MCs. Moreover, we demonstrated that pre-conditioning with high glucose caused rapid generation of mesangial oxidative stress within several minutes of Ang II-stimulation. Such Ang II-induced generation of oxidative stress under high glucose condition resulted from AT1R effect, since AT1R inhibitor apparently restored the ROS level to a normal range.

Ang II exposure has already been known to cause mesangial cell damage or apoptosis^19^. As discussed earlier, MCs are not merely supporting tissues which sustain glomerular structure, but they can also contract capillaries or function as phagocytes. Mesangial damage has been known to lead to many glomerular diseases, including diabetic nephropathy^20,21^. However, the exact physiological mechanism of mesangial damage in diabetic nephropathy is not yet known, even though diabetic nephropathy is the leading cause of end-stage renal disease^22^. This study demonstrated a potential mechanism (*i*.*e*. ROS production in MCs) behind the development of diabetic nephropathy under mildly-elevated serum glucose levels, which is further augmented by Ang II stimulation. This theory is supported by a previous study which showed that co-administration of high levels of glucose and Ang II stimulation under chronic condition activated MC inflammation via toll-like receptor 4 signaling^23^. In addition to the findings of this previous report, our data demonstrated that oxidative stress was induced in MCs shortly after the stimulation of Ang II. In addition, our data indicated that if such Ang II stimulation persisted for more than several hours, MC oxidative stress could be induced even under the condition of only mildly elevated glucose levels.

Based on these findings that glucose and Ang II stimulation may play synergistic effect on ROS production, an important therapeutic strategy for diabetic nephropathy has been suggested wherein it would be necessary to maintain the serum glucose level within a normal range. In addition, suppressing the renin-angiotensin-aldosterone axis by Ang II receptor blockers (ARBs) would be effective in preventing the progression of mesangial damage. More specifically, based on the result shown in Fig. 4, AT1R-receptor blockers, such as olmesartan, suppressed the mesangial oxidative stress. This result corroborated with the previous studies that demonstrated the advantage of AT1R antagonist, such as olmesartan, losartan, or irbesartan in therapeutic management of the hypertensive patients with type-2 diabetes^24–27^. Considered together with our findings, AT1R antagonists would be beneficial both for controlling hypertension by blocking AT1R-mediated vessel contraction and for preventing end-stage renal disease by suppressing the AT1R-mediated mesangial oxidative stress. In hypertensive patients with type-2 diabetes, administration of AT1R antagonists is highly recommended, along with the management of serum glucose level.

As a limitation of this study, the exact molecular pathway correlating high glucose level and mesangial Ang II-induced production of ROS has not been identified yet. Based on several previous reports, TLR4 and NADPH oxidase 4 could be among the promising candidates; however, we need to conduct additional experiments to validate this speculation^28–31^.

## Conclusions

High glucose level in the surrounding extracellular fluid augmented mesangial Ang II-induced ROS production via AT1R leading to oxidative stress. Both glucose and Ang II exert synergistic effects on ROS production. Under the condition of high glucose level, Ang II stimulation rapidly activated ROS production in MCs. When MCs were exposed to Ang II under chronic condition for more than several hours, even mildly-elevated glucose level could increase the ROS production in them. In practice, not only adjusting the serum glucose level of the DM patients, but also suppressing the excessive activity of RAA axis could be a good strategy to prevent the mesangial damage in hyperglycemia-induced glomerular diseases.

## Supplementary information

Supplementary Table 1

## Data Availability

All relevant data are provided within the paper and its supporting file (Supplementary Table 1).
